# Prediction of the Miscibility of PBAT/PLA Blends

**DOI:** 10.3390/polym13142339

**Published:** 2021-07-16

**Authors:** Shen Su

**Affiliations:** 1 Department of Circular and Bio-Based Plastics, Fraunhofer UMSICHT, Institute for Environment, Safety and Energy Technology, Osterfelder Str. 3, 46047 Oberhausen, Germany; shen.su@umsicht.fraunhofer.de or shen.su@ruhr-uni-bochum.de; Tel.: +49-208-8598-1422; 2 Department of Mechanical Engineering, Ruhr-University Bochum, Universitaetsstr. 150, 44780 Bochum, Germany

**Keywords:** Flory–Huggins, free energy of mixing, glass transition temperature, group contribution, molecular weight, miscibility prediction, PBAT, PLA, simulation, solubility parameter

## Abstract

Designing polymer structures and polymer blends opens opportunities to improve the performance of plastics. Blending poly(butylene adipate-co-terephthalate) (PBAT) and polylactide (PLA) is a cost-effective approach to achieve a new sustainable material with complementary properties. This study aimed to predict the theoretical miscibility of PBAT/PLA blends at the molecular level. First, the basic properties and the structure of PBAT and PLA are introduced, respectively. Second, using the group contribution methods of van Krevelen and Hoy, the Hansen and Hildebrand solubility parameters of PBAT and PLA were calculated, and the effect of the molar ratio of the monomers in PBAT on the miscibility with PLA was predicted. Third, the dependence of the molecular weight on the blend miscibility was simulated using the solubility parameters and Flory–Huggins theory. Next, the glass transition temperature of miscible PBAT/PLA blends, estimated using the Fox equation, is shown graphically. According to the prediction and simulation, the blends with a number-average molecular weight of 30 kg/mol for each component were thermodynamically miscible at 296 K and 463 K with the possibility of spinodal decomposition at 296 K and 30% volume fraction of PBAT. This study contributes to the strategic synthesis of PBAT and the development of miscible PBAT/PLA blends.

## 1. Introduction

Physical blending is an economical approach to achieve complementary material properties without developing a new polymer. Poly(butylene adipate-co-terephthalate) (PBAT) and polylactide (PLA) currently account for 13.5 and 18.8 percent of global bioplastic production capacity, respectively [1]. Blends made of commercially available PBAT and PLA tend to macrophase-separate and exhibit two glass transition temperatures (*T_g_*) [2,3,4]. Therefore, unmodified PBAT/PLA blends have poor miscibility.

Experimentally, PBAT/PLA blends are often prepared by melt blending. At an elevated temperature for a sufficiently long time, transesterification can take place between the two polyesters, resulting in enhanced miscibility [5]. Two criteria often used to evaluate the blend miscibility [6,7,8] include (1) phase morphology: whether it is homogeneous down to the molecular level; (2) glass transition temperature: whether a binary blend exhibits a single *T_g_*. The morphological investigation depends on the measuring points of the samples in scanning electron microscopy (SEM). A blend may not be truly miscible at the molecular level, even though it shows a homogeneous phase if examined on sufficiently large length scales [6]. Thermal analysis using differential scanning calorimetry (DSC) can only determine the miscibility of polymer blends with well-separated *T_g_* values [9]. Furthermore, different heating rates can lead to a variation in *T_g_* values to some extent. Therefore, both the blend preparation and the analysis can practically affect the actual blend miscibility.

To exclude the experimental influences, a theoretical study of the intrinsic miscibility is necessary. The concept of group contributions has been applied to calculate the solubility parameters and the blend miscibility [10]. According to the literature research, Dil et al. indicate that PBAT and PLA have the Hildebrand solubility parameters (HiSP) of 22.2 MPa^1/2^ and 21.9 MPa^1/2^, respectively [11]. Ding et al. report that the δ (PBAT) and δ(PLA) are 21.9 MPa^1/2^ and 20.7 MPa^1/2^, respectively [12]. As a result, the difference of solubility parameters *δ*(PBAT-PLA) is 0.3 or 1.2 MPa^1/2^. Materials with similar HiSP have a high affinity with each other [13]. However, the PBAT/PLA blend miscibility is still difficult to determine directly from the difference in solubility parameters. One reason for this is the irregular structure of PBAT molecules that consist of two randomly arranged monomers (BA and BT). Their molar ratio can differ due to the synthesis and degradation. If PBAT were degraded, the resulting lower molecular PBAT-chains might have different ratios of BA and BT. Another reason is that the intermolecular interaction depends on the molecular weights of both polymers, blend ratio, and temperature [14]. To the author’s best knowledge, only Park et al. [15] have studied the miscibility of blends (PETG/PLA) with some similarity in molecular structure to PBAT/PLA blends from the theoretical aspect. Therefore, PBAT/PLA blends need more investigation regarding the solubility parameters and miscibility from the molecular level and the thermodynamic aspect. A good understanding of the intrinsic miscibility is not only of academic interest but also contributes to designing miscible PBAT/PLA blends without adding expensive additives.

This study aimed to explore the dependence of the PBAT structure and the molecular weights of PBAT and PLA with different weight ratios on the blend miscibility. First, group contributions theories were used to estimate the effect of the molar ratio of the two monomers in PBAT on the solubility parameter and the difference of solubility parameter between both polymers. Second, the Flory–Huggins model was used to establish phase diagrams and spinodal curves. Furthermore, the composition-dependent glass transition of miscible PBAT/PLA blends was estimated. The first novelty was to find a correlation between the molar ratios of the two monomers in PABT and the solubility parameter between PBAT and PLA. The second novelty was to simulate the phase diagrams and spinodal curves of PBAT/PLA blends, taking into account parameters including temperature, molecular weights, and the component ratios, as well as the calculated solubility parameters.

## 2. Materials

**Figure 1:**
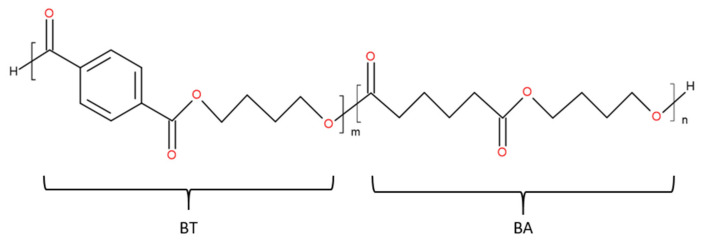
Chemical structure of PBAT.

Poly(butylene adipate-co-terephthalate) (PBAT) (Figure 1) is a fully biodegradable polyester with two types of dimers: BT and BA. One dimer is the rigid section consisting of 1,4-butanediol and terephthalic acid monomers. The other dimer is the flexible section consisting of 1,4-butanediol and adipic acid monomers [16], resulting in high flexibility and high ductility [4]. The largest manufacturer, BASF (Ludwigshafen, Germany), produces PBAT under the brand name Ecoflex^®^ (e.g., Ecoflex F Blend C1200). This polymer possesses a density of 1.26 g/mol, a number average molecular weight (*M_n_*) of 52.1 kg/mol, and a polydispersity index of 2.0.

**Figure 2:**
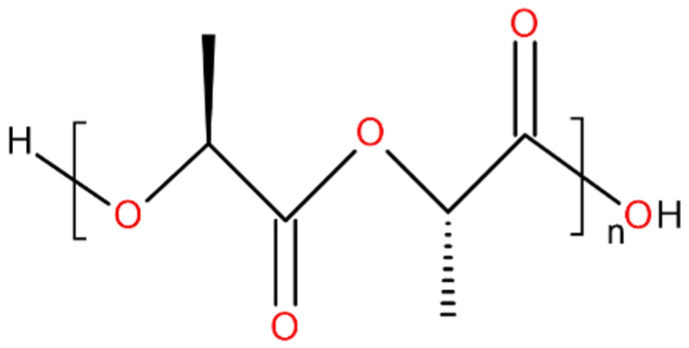
Chemical structure of PLLA.

Poly(lactic acid) or polylactide (PLA) is a biodegradable and renewable aliphatic polyester [17]. Poly(lactic acid) is produced by direct polycondensation of lactic acid. Polylactide can be produced by ring-opening polymerization of cyclic lactide. PLA possesses two optical active and crystallizable isomeric forms: PDLA and PLLA (Figure 2). PLA exhibits many favorable features such as high modulus of elasticity, high strength and high transparency (in the amorphous state), and good processability [18]. The largest manufacturer, NatureWorks (Minnetonka, MN, USA), produces PLA under the trade name Ingeo^TM^ (for example, Ingeo^TM^ Biopolymer 2003D). This polymer has a density of 1.24 g/mol, a number average molecular weight (*M_n_*) of 127.0 kg/mol, a polydispersity index of 1.6, and a D-isomer content of approximately 4.4%.

## 3. Prediction of Solubility Parameters

The basis of a molecular group contribution method is additivity. This assumes that the physical property of a polymer is calculable by the additive contributions from the individual structural and functional groups in its repeating unit. Using a group contribution method enables us to establish a correlation between the chemical structure of polymers and their interaction. The term “solubility parameter” quantifies the intermolecular interaction.

Coleman et al. has reported a group contribution method to calculate the one-dimensional Hildebrand solubility parameter (HiSP) for polymers with *T_g_* values below room temperature [19]. However, PLA possesses a glass transition temperature higher than room temperature (approx. 60 °C). Therefore, Coleman’s method was inappropriate in this miscibility study of PBAT/PLA blends. Group contribution methods developed by van Krevelen [10] and Hoy [20] were used to estimate the three-dimensional HSP and one-dimensional HiSP for polymers. Compared with the real system, the group contribution methods neglect the possible reactions between the blend components, such as transesterification while melt blending, which may change the molecular structures.

In this section, these two different group contribution methods were applied to calculate the solubility parameters of PBAT and PLA. As mentioned in the Introduction, PBAT can consist of different ratios of its monomers (BA and BT). Furthermore, the sequence in the chain may change due to, e.g., polymer degradation. Considering these variations, the estimation of its solubility parameter was performed using three different assumptions:

(1) PBAT (alternating): alternating arrangement with a molar ratio of 1/1.
(2) (PBT): only monomers of BT constitute the polymer.
(3) (PBA): only monomers of BA constitute the polymer.

### 3.1. Solubility Parameter Calculation According to van Krevelen’s Method

The group contributions *F_di_*, *F_pi_*, and *E_hi_* [10] are applied to calculate HSP, including dispersion interactions (*δ_d_*), polar interactions (*δ_p_*), and hydrogen bond interactions (*δ_h_*), as well as the total Hildebrand solubility parameters (*δ_t_* or HiSP). The following equations are used for the calculation:

(1) $$\delta_{d}=\frac{\sum F_{di}}{V}$$

(2) $$\delta_{p}=\frac{\sqrt{\sum F_{pi}^{2}}}{V}$$

(3) $$\delta_{h}=\sqrt{\frac{\sum E_{hi}}{V}}$$

(4) $$\delta_{t}=\sqrt{\delta_{d}^{2}+\delta_{P}^{2}+\delta_{h}^{2}}$$

where *F_di_* represents the group contributions of type *i* to the dispersion component *F_d_* of the molar attraction constant; *F_pi_* represents the group contributions to the polar component *F_p_*; *E_hi_* is the hydrogen-bonding energy per structural group *i*; *V* is the molar volume. Details of the calculation are described in the Supplementary Material on the sheet “van Krevelen”. The calculated HSP and HiSP using van Krevelen’s method are listed (Table 1).

**Figure 3:**
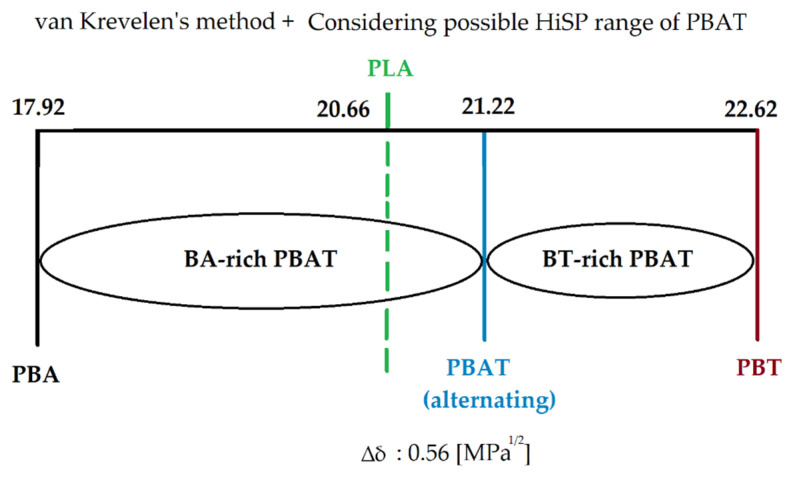
Values and ranges of calculated solubility parameters using van Krevelen’s method.

Due to the random arrangement of the copolymers BT and BA and possible different molar ratios, the solubility parameters for each segment is different. In the case of a BT-rich PBAT (molar ratio of BT ≥ 50%), the HiSP of PBAT was between 21.22 and 22.62, which are the values of alternating PBAT with BT/BA = 1/1 and PBT. In the case of a BA-rich PBAT (molar ratio of BA ≥ 50%), the HiSP of PBAT was between 17.92 and 21.22, the values of PBA and alternating PBAT with BA/BT =1/1. The calculated HiSP of PBAT (alternating) and PLA was 21.22 [MPa^1/2^] and 20.66 [MPa^1/2^], respectively, so the difference of total solubility parameter ∆δ between them was 0.56 [MPa^1/2^]. Since the HiSP of PLA was in the range of BA-rich PBAT (Figure 3), varying the molar ratio of the monomers in PBAT may lead to a smaller difference in solubility parameters and an increase in the intermolecular interaction between PBAT and PLA.

### 3.2. Solubility Parameter Calculation According to Hoy’s Method

The methods of van Krevelen and Hoy, based on the same basic assumption of additivity, have the same order of accuracy. These two are of the same order of accuracy for predicting the solubility parameters [10]. However, van Krevelen and Hoy have applied different ways of calculation. Van Krevelen’s method first predicts the partial solubility parameters, which are then used to calculate the total solubility parameter [10,21]. In contrast, Hoy’s method first determines the total solubility parameter using the molar attraction functions and auxiliary equations. From the total, the three partial ones are calculated using additive molar functions and expressions for the components [20,22]. The equations used in Hoy’s method are below.

(5) $$\alpha \left(P\right)=777\Delta_{T}\left(P\right)/V$$

(6) $$n=0.5/\Delta_{T}\left(P\right)$$

(7) $$\delta_{t}=\left(F_{i}+\frac{B}{\bar{n}}\right)/V$$

(8) $$\delta_{P}=\delta_{t}{\left(\frac{1}{\alpha \left(P\right)}\frac{F_{P}}{F_{t}+B/\bar{n}}\right)}^{1/2}$$

(9) $$\delta_{h}=\delta_{t}{\left[\left(\alpha \left(P\right)-1/\alpha \left(P\right)\right)\right]}^{1/2}$$

(10) $$\delta_{d}={\left(\delta_{t}^{2}-\delta_{P}^{2}-\delta_{h}^{2}\right)}^{1/2}$$

where *α(P)* is the molecular aggregation number of a polymer; ∆*_T_(P)* is the Lydersen correction for polymers derived by Hoy; *V* is the molar volume; B is a base value of 277; *F_t_* is the molar attraction function; *F_P_* is the polar component of molar attraction function; *n* is the molecular aggregation number. For explanations of variables used before, see Section 3.1 “van Krevelen’s method”.

The three-dimensional HSP and the overall one-dimensional HiSP were calculated (Table 2). (For details of the calculation, see the Supplementary Material on the sheet “Hoy”).

**Figure 4:**
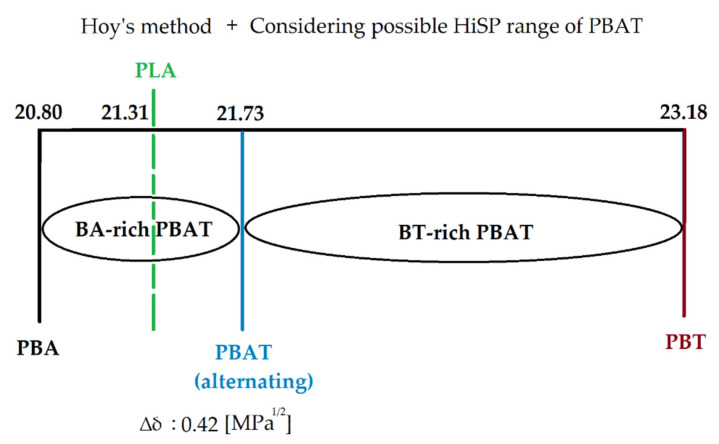
Values and ranges of calculated solubility parameters using Hoy’s method.

From the table above, it can be seen that BA-rich PBAT had a HiSP in the range of 20.8–21.73, while BT-rich PBAT possessed a HiSP in the range of 21.73 to 23.18 [MPa^1/2^]. The calculated HiSP for PBAT and PLA was 21.73 [MPa^1/2^] and 21.31 [MPa^1/2^], respectively, indicating a solubility parameter difference of 0.42 [MPa^1/2^]. Furthermore, Hoy’s method shows the same tendency as van Krevelen’s method: that the HiSP of PLA was in the range of the values of BA-rich PBAT (Figure 4).

According to the two group contribution methods, the mean value of the difference in calculated total solubility parameters between alternating PBAT and PLA was 0.49 MPa^1/2^. The two different methods show consistency in the total solubility parameter and that PLA was in the range of the one of BA-rich PBAT. Furthermore, the same tendency of the solubility parameter can be seen in both methods:

δ(PBA) < δ(PLA) < δ(alternating PBAT) < δ(PBT)

## 4. Simulation of the Blend Miscibility

Based on the solubility parameters, the miscibility of PBAT/PLA blends can be further studied from the aspect of thermodynamics. The thermodynamic criterion of solubility of two dissimilar components is described by the equation:

(11) $$\Delta G_{M}=\Delta H_{M}-T\Delta S_{M}$$

where ∆*G_m_* is the free energy of mixing; the ∆*H_M_* is the enthalpy of mixing (heat of mixing); *T* is the absolute temperature; ∆*S_M_* is the entropy of mixing.

A negative value of ∆*G_m_* is generally required to obtain a miscible system. For low molecular weight materials, an increasing temperature generally results in an increase in miscibility as the *T*∆*S_M_* term increases, thus driving *G_M_* to be more negative [8,23]. However, both PBAT and PLA are high molecular weight molecules, implying that the negative contribution from the *T*∆*S_M_* term is small. An equation based on the Flory–Huggins theory correlates the miscibility of a polymer blend with several parameters [24].

(12) $$\frac{\Delta G_{M}}{RTV}=\frac{\phi_{1}}{V_{1}}ln\phi_{1}+\frac{\phi_{2}}{V_{2}}ln\phi_{2}+\frac{\phi_{1}\phi_{2}}{RT}{\left(\delta_{1}-\delta_{2}\right)}^{2}$$

where ∆*G_M_* is the free enthalpy of mixing; R the gas constant of 8.31 kg·m^2^/s^2^·mol·K; *T* the absolute temperature; *V* the volume of the system; *ϕ*_1_ and *ϕ*_2_ the volume fraction of component, respectively; *δ*_1_ and *δ*_2_ are the solubility parameters; *V*_1_ and *V*_2_ are the molar volumes, respectively. Since the densities of PBAT and PLA are very close, for simplicity, we assumed that both polymers had the same density of 1.25 g/mol. Based on this assumption, for PBAT/PLA blends, the volume fraction is equal to the mass fraction.

**Figure 5:**
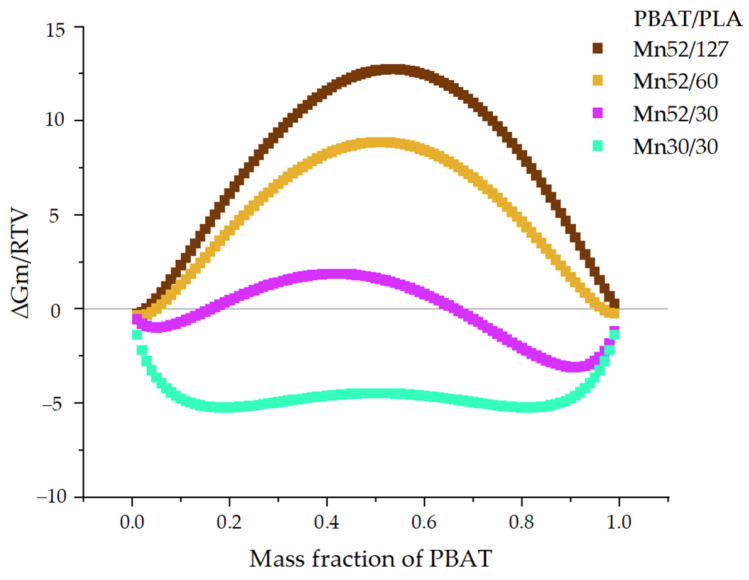
Phase diagram of PBAT/PLA blends with various molecular weights at 296 K.

The phase diagram (Figure 5) offers a simulation of the PBAT/PLA blend miscibility with assumptions at room temperature (296 K, approximately 23 °C), which is important for the blend preparation by solution blending. PBAT structure was alternating; the HiSP difference was 0.49 MPa^1/2^; the density of both polymers was 1.25 kg/mol; the blend had the molecular weights: *M_n_*52/127, *M_n_*52/60, *M_n_*52/30, and *M_n_*30/30. As an example, *M_n_*52/127 implies *M_n_*(PBAT) is 52 kg/mol and *M_n_*(PLA) is 127 kg/mol, respectively. Details of the calculation are in the Supplementary Material on the sheet “Flory–Huggins”.

From the phase diagram, the curve of the PBAT/PLA blends *M_n_*52/127 with the respective molecular weight of 52 and 127 kg/mol is above the zero line, implying blend immiscibility made of the commercially available polymers. A similar trend is shown by the curve *M_n_*52/60, representing the blend made of the original PBAT and PLA with molecular weights of 60 kg/mol. When the molecular weight of PLA was reduced to 30 kg/mol, *M_n_*52/30 showed an interesting curve: in the region of low PBAT content (<15 wt.%), the value of free energy of mixing was slightly negative; this was followed by the curve with a PBAT mass fraction of 15–70 wt.%. Starting with a PBAT content of approximately 70 wt.%, the curve was in the negative region again. The PBAT/PLA blends with the molecular weight of 30 kg/mol (*M_n_*30/30) showed negative values of the free energy of mixing in the whole range. However, it can be seen that in the middle range (40–60 wt.% PBAT), this curve exhibited a slight increase, while the curve shows even lower negative values at the PBAT/PLA ratio of about 20/80 or 80/20. Generally, a lower negative value would lead to a higher probability of miscibility.

**Figure 6:**
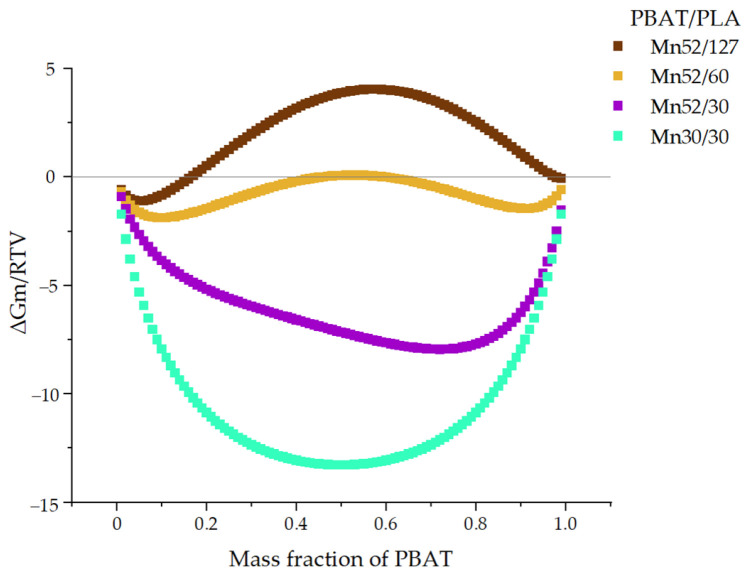
Phase diagram of PBAT/PLA blends with various molecular weights at 463 K.

Since PBAT/PLA blends are often melt-blended at an elevated temperature, the blend miscibility at 463 K, approx. 190 °C, was simulated (Figure 6).

Even at this higher temperature, *M_n_*52/127 (PBAT/PLA blends consisting of original commercial polymers) showed a curve mostly close to or above the zero line, implying poor miscibility. With the decreased molecular weights of PLA, the curve of *M_n_*52/60 displays a maximum slightly above the zero line (when PBAT content is approx. 50–60 wt.%) and two minima slightly below the zero line (when PBAT content is around 10 wt.% or 90 wt.%), indicating a relatively unstable state of miscibility. A small fluctuation of the temperature or composition may change the theoretical miscibility. When the molecular weights of PLA were 30 kg/mol, *M_n_*52/30 blends showed negative values of the free energy of mixing in the whole range, indicating the blend should be miscible at 463 K. The blends with an *M_n_* of 30 kg/mol for each component demonstrated even lower values, indicating thermodynamically miscibility in the molten state at 463 K.

**Figure 7:**
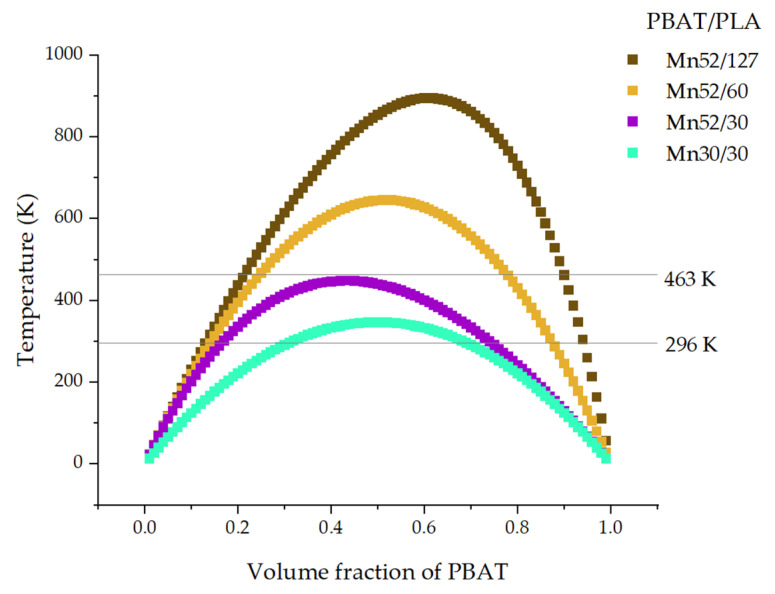
Spinodal curves for PBAT/PLA blends.

It is concluded that the mixing behavior depends strongly on the molecular weights of both components, their ratios, and the temperature. As shown in both phase diagrams, the miscibility of PBAT/PLA generally increases with decreasing molecular weights. At 296 K, the blends *M_n_*52/127, *M_n_*52/60, and *M_n_*52/30 each showed a maximum positive value of ∆*G_M_* in the middle range (PBAT content: approx. 50 wt.%) in the curves, indicating poor miscibility of these blends, especially at the blend ratio of 50/50. Generally, in addition to the negative value of ∆*G_M_*, the second derivative of ∆*G_M_* with respect to the volume fraction of the second blend components was a necessary condition of the blend miscibility. At 296 K, the blends *M_n_*30/30 showed negative values of ∆*G_M_* in the whole range of compositions while displaying two minima (at the ratio of about 10/90 and 90/10) and one maximum (at the ratio of 50/50). According to the second derivative of ∆*G_M_* (eq.12 = 0, at 30% volume fraction of PBAT), spinodal decomposition can occur at 296 K due to the negative value of free energy of mixing but beginning of the convex curve of the spinodal (Figure 7).

At 463 K (e.g., for melt-blending), the curve of the blends *M_n_*30/30 shows negative values in the entire range of compositions indicating good miscibility. Even the blends *M_n_*52/30 would be miscible in the molten state while melt-blending at 463 K. After that, if the PBAT/PLA blends *M_n_*52/30 were stored at a lower temperature between −28 °C and 61 °C (corresponding to the *T_g_* values of both polymers) for a sufficiently long time, the PBAT chains would behave like rubber. For this reason, the blends *M_n_*52/30 could change the miscibility from miscible in the molten state to partially miscible or immiscible at a lower temperature after a long enough time. However, the PBAT/PLA with an *M_n_* of 30 kg/mol for each component should be miscible both at 296 K and 463 K, according to the simulation.

## 5. Estimation of the Glass Transition Temperature

*T_g_* can reflect whether a polymer blend is completely miscible. A fully miscible PBAT/PLA blend exhibits a single *T_g_*. Generally, the *T_g_* value of binary miscible PBAT/PLA blends is predictable based on the Fox equation:

(13) $$\frac{1}{T_{g}\left(\mathrm{Blend}\right)}=\frac{m_{\mathrm{PBAT}}}{T_{g}\left(\mathrm{PBAT}\right)}+\frac{1-m_{\mathrm{PBAT}}}{T_{g}\left(\mathrm{PLA}\right)}$$

where *T_g_*(Blend) is the predicted glass transition temperature of the miscible PBAT/PLA blend; *m*_PBAT_ is the mass (weight) fraction of PBAT; *T_g_*(PBAT) and *T_g_*(PLA) are the glass transition temperature of neat PBAT and neat PLA, respectively.

**Figure 8:**
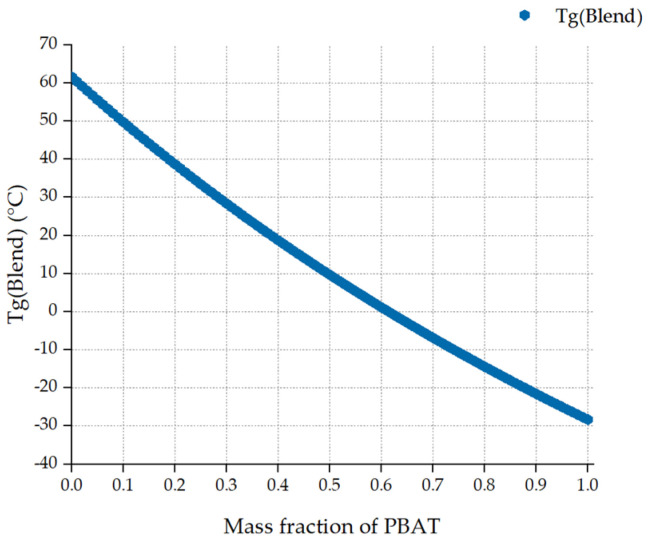
Glass transition temperature of miscible PBAT/PLA blends.

The value of *T_g_*(PBAT) and *T_g_*(PLA) was −28.3 °C and 61.6 °C, respectively [4]. For details of the *T_g_*(Blend) calculation, see the Supplementary Material on the sheet “Fox”. The composition-dependent *T_g_* of miscible PBAT/PLA blends is shown graphically (Figure 8).

*T_g_*(Blend) tends to decrease with an increasing mass fraction of PBAT. The glass transition temperature is slightly below 40 °C for PBAT/PLA (20/80). This value is about 10 °C for PBAT/PLA (50/50). Moreover, this value decreases to approximately −15 °C when the ratio of PBAT/PLA is 80/20. To the author’s best knowledge, the glass transition temperatures have not been studied for fully miscible PBAT/PLA blends without compatibilizers. Unmodified PBAT/PLA blends with a wide range of ratios (0/100, 10/90, … 90/10, 100/0) have been reported to have two almost unchanged glass transition temperatures at about −30 °C and 61 °C, which correspond to the *T_g_* of neat PBAT and PLA in the DSC [4].

## 6. Conclusions

In this study, the blend miscibility of PBAT/PLA blends was predicted. The solubility parameters calculated using the group contribution methods of van Krevelen and Hoy had a mean difference of 0.49 MPa^1/2^ between alternating PBAT and PLA. To a certain extent, a higher affinity would be possible between the two polymers when the monomers BA and BT reach a molar ratio exceeding 1 to 1. In this way, the structural optimization of PBAT will fundamentally improve the solubility of PBAT and PLA. Furthermore, a simulation of the miscibility of PBAT/PLA blends was established by using the calculated HiSP and different parameters. According to the simulation, the state of a PBAT/PLA blend can vary from immiscible to miscible, depending strongly on the molecular weights and weight ratio of both polymers at a constant temperature. Generally, the higher the molecular weights, the lower the predicted probability of the blend miscibility. Another tendency is that the higher the temperature, the higher the probability of the blend miscibility. The blends *M_n_*52/30 displayed negative values in the whole range of compositions at 463 K. If *M_n_*52/30 were melt-blended and then stored at a temperature above the *T_g_* of PBAT (−28 °C, approx. 245 K) for enough long time, the miscibility could change from miscible to partially miscible or immiscible, due to the mobility of PBAT chains. The blends *M_n_*30/30 showed negative values of ∆*G_M_* both at 296 K and 463 K, according to the simulation. However, spinodal decomposition of *M_n_*30/30 can appear at 296 K (at 30% volume fraction of PBAT) due to the negative value of ∆*G_M_* and the curvature of the spinodal. Moreover, the glass transition temperature of miscible PBAT/PLA blends was calculated using the Fox equation. A single *T_g_* would show at about 40 °C, 10 °C, and −15 °C for PBAT/PLA blends with the composition of (20/80), (50/50), and (80/20), respectively. This study gives the theoretical prediction of the miscibility for PBAT/PLA blends. The next scientific challenge will be the experimental discovery of to what extent the theoretical prediction is consistent with the practical results, especially the molecular weight-dependent miscibility of PBAT and PLA.

## Figures and Tables

**Table 1:** Calculated solubility parameters using van Krevelen’s method.

Polymer Type	*δ_d_* [MPa^1/2^]	*δ_p_* [MPa^1/2^]	*δ_h_* [MPa^1/2^]	HiSP [MPa^1/2^]
alternating PBAT	18.21	5.89	9.17	21.22
PBT	19.63	6.19	9.37	22.62
PBA	14.99	5.00	8.45	17.92
PLA	15.33	8.44	10.98	20.66

**Table 2:** Calculated solubility parameters using Hoy’s method.

Polymer Type	*δ_d_* [MPa^1/2^]	*δ_p_* [MPa^1/2^]	*δ_h_* [MPa^1/2^]	HiSP [MPa^1/2^]
alternating PBAT	15.94	11.43	9.35	21.73
PBT	15.64	12.82	11.32	23.18
PBA	16.13	10.44	7.98	20.80
PLA	14.02	12.73	9.77	21.31

## Data Availability

For details of the data used in this paper, see the supplementary material.

## Supplementary Materials

The following are available online at https://www.mdpi.com/article/10.3390/polym13142339/s1, Excel table with the sheets of “vanKrevelen”, “Hoy”, “Flory–Huggins” and “Fox”.

## References

[1] European Bioplastics e.V Bioplastics Market Data. https://www.european-bioplastics.org/market/.

[2] Liu T.-Y., Lin W.-C., Yang M.-C., Chen S.-Y. (2005). Miscibility, thermal characterization and crystallization of poly(l-lactide) and poly(tetramethylene adipate-co-terephthalate) blend membranes. Polymer.

[3] Farsetti S., Cioni B., Lazzeri A. (2011). Physico-Mechanical Properties of Biodegradable Rubber Toughened Polymers. Macromol. Symp..

[4] Su S., Duhme M., Kopitzky R. (2020). Uncompatibilized PBAT/PLA Blends: Manufacturability, Miscibility and Properties. Materials.

[5] Kim Y.J., Park O.O. (1999). Miscibility and Biodegradability of Poly(Butylene Succinate)/Poly(Butylene Terephthalate) Blends. J. Polym. Environ..

[6] Arrighi V., Cowie J.M., Fuhrmann S., Youssef A., Isayev A.I. (2010). Miscibility Criterion in Polymer Blends and its Determination. Encyclopedia of polymer blends. Volume 1, Fundamentals.

[7] Imre B., Renner K., Pukanszky B. (2014). Interactions, structure and properties in poly(lactic acid)/thermoplastic polymer blends. Express Polym. Lett..

[8] Hamad K., Kaseem M., Ayyoob M., Joo J., Deri F. (2018). Polylactic acid blends: The future of green, light and tough. Prog. Polym. Sci..

[9] Fekete E., Földes E., Pukánszky B. (2005). Effect of molecular interactions on the miscibility and structure of polymer blends. Eur. Polym. J..

[10] van Krevelen D.W., Nijenhuis K.t. (2009). Properties of Polymers: Their Correlation with Chemical Structure; Their Numerical Estimation and Prediction from Additive Group Contributions/D.W. van Krevelen.

[11] Dil E.J., Carreau P.J., Favis B.D. (2015). Morphology, miscibility and continuity development in poly(lactic acid)/poly(butylene adipate-co-terephthalate) blends. Polymer.

[12] Ding Y., Feng W., Huang D., Lu B., Wang P., Wang G., Ji J. (2019). Compatibilization of immiscible PLA-based biodegradable polymer blends using amphiphilic di-block copolymers. Eur. Polym. J..

[13] Hansen C.M. (2007). Hansen Solubility Parameters: A User’s Handbook.

[14] Nedoma A.J., Robertson M.L., Wanakule N.S., Balsara N.P. (2008). Measurements of the Composition and Molecular Weight Dependence of the Flory−Huggins Interaction Parameter. Macromolecules.

[15] Park J.Y., Hwang S.Y., Yoon W.J., Yoo E.S., Im S.S. (2012). Compatibility and physical properties of poly(lactic acid)/poly(ethylene terephthalate glycol) blends. Macromol. Res..

[16] Kijchavengkul T., Auras R., Rubino M., Selke S., Ngouajio M., Fernandez R.T. (2010). Biodegradation and hydrolysis rate of aliphatic aromatic polyester. Polym. Degrad. Stab..

[17] Farah S., Anderson D.G., Langer R. (2016). Physical and mechanical properties of PLA, and their functions in widespread applications—A comprehensive review. Adv. Drug Deliv. Rev..

[18] Su S., Kopitzky R., Tolga S., Kabasci S. (2019). Polylactide (PLA) and Its Blends with Poly(butylene succinate) (PBS): A Brief Review. Polymers.

[19] Coleman M.M., Serman C.J., Bhagwagar D.E., Painter P.C. (1990). A practical guide to polymer miscibility. Polymer.

[20] Hoy K.L. (1989). Solubility Parameter as a Design Parameter for Water Borne Polymers and Coatings. J. Coat. Fabr..

[21] Stefanis E., Panayiotou C. (2008). Prediction of Hansen Solubility Parameters with a New Group-Contribution Method. Int. J. Thermophys..

[22] Kitak T., Dumičić A., Planinšek O., Šibanc R., Srčič S. (2015). Determination of Solubility Parameters of Ibuprofen and Ibuprofen Lysinate. Molecules.

[23] Robeson L.M. (2007). Polymer Blends: A Comprehensive Review.

[24] Jost V., Kopitzky R. (2015). Blending of Polyhydroxybutyrate-co-valerate with Polylactic Acid for Packaging Applications—Reflections on Miscibility and Effects on the Mechanical and Barrier Properties. Chem. Biochem. Eng. Q..

